# Preventing acute kidney injury in high-risk patients by temporarily discontinuing medication – an observational study in general practice

**DOI:** 10.1186/s12882-019-1636-z

**Published:** 2019-12-04

**Authors:** Suzanne J. Faber, Nynke D. Scherpbier, Hans J. G. Peters, Annemarie A. Uijen

**Affiliations:** Department of Primary and Community Care, Radboud University Nijmegen Medical Centre, Nijmegen, The Netherlands

**Keywords:** Dehydration, Deprescribing, Acute kidney injury, Chronic kidney disease, General practice

## Abstract

**Background:**

Elderly, patients with chronic kidney disease (CKD) and patients with heart failure who continue using renin-angiotensin-aldosterone-system (RAAS) inhibitors, diuretics, or non-steroidal-anti-inflammatory drugs (NSAIDs) during times of fluid loss have a high risk of developing complications like acute kidney injury (AKI). The aim of this study was to assess how often advice to discontinue high-risk medication was offered to high-risk patients consulting the general practitioner (GP) with increased fluid loss. Furthermore, we assessed the number and nature of the complications that occurred after GP consultation.

**Methods:**

We performed a cross-sectional study with patients from seven Dutch general practices participating in the Family Medicine Network between 1 and 6-2013 and 1-7-2018. We included patients who used RAAS-inhibitors, diuretics, or NSAIDs, and had at least one of the following risk factors: age ≥ 70 years, CKD, or heart failure. From this population, we selected patients with a ‘dehydration-risk’ episode (vomiting, diarrhoea, fever, chills, or gastrointestinal infection). We manually checked their electronic patient files and assessed the percentage of episodes in which advice to discontinue the high-risk medication was offered and whether a complication occurred in 3 months after the ‘dehydration-risk’ episode.

**Results:**

We included 3607 high-risk patients from a total of 44.675 patients (8.1%). We found that patients were advised to discontinue the high-risk medication in 38 (4.6%) of 816 ‘dehydration-risk’ episodes. In 59 of 816 episodes (7.1%) complications (mainly AKI) occurred.

**Conclusions:**

Dutch GPs do not frequently advise high-risk patients to discontinue high-risk medication during ‘dehydration-risk’ episodes. Complications occur frequently. Timely discontinuation of high-risk medication needs attention.

## Introduction

Dehydration is a widespread and significant problem, that is more prevalent in infants, elderly and athletes [1–3]. In healthy patients, dehydration can be resolved quickly by oral rehydration. However, in some cases, dehydration can lead to intravascular volume depletion, which can cause severe complications such as acute kidney injury (AKI) or hypotension [4, 5].

Patients with pre-existent chronic kidney disease (CKD), heart failure, and elderly patients have an increased risk of these complications [6], especially when suffering from concurrent illnesses that cause fluid loss such as vomiting, diarrhoea and fever [7]. In these groups, volume regulation and water and sodium homeostasis is impaired.

When patients also use drugs that inhibit the renin-angiotensin-aldosterone-system (RAAS -inhibitors), non-steroidal anti-inflammatory drugs (NSAIDs), or diuretics, this risk is even higher [6, 8]. In patients who use RAAS-inhibitors, water and sodium retention is impaired, causing a decrease in blood pressure and renal blood flow. RAAS-inhibitors and NSAIDs also impair glomerular haemodynamics, leading to a diminished glomerular filtration pressure and renal function [9–12]. Patients who use diuretics, have a higher risk of suffering from pre-renal acute kidney injury [13]. This is due to the fact that diuretics decrease extracellular volume by increasing diuresis. A mild concurrent illness with additional fluid loss such as a period of fever, or vomiting can trigger AKI in these patients.

This ‘cocktail’ of dangerous factors (fragile patients using RAAS-inhibitors, NSAIDS or diuretics combined with dehydration) has been described in multiple case reports [6, 14, 15], and retrospective studies [16–18]. In almost all of these cases, this cocktail ultimately led to severe reversible kidney injury or severe hypotension with haemodynamic collapse. The main causes of dehydration were vomiting and diarrhoea.Guidelines recommend to (consider to) temporarily discontinue high-risk medication in patients at risk for dehydration to prevent complications [4, 19, 20].

The extent and impact of these situations in general practice is unknown. In this research we aimed to determine the number of patients at risk in general practice, guideline adherence and the incidence of complications.

## Methods

### Design and setting

We performed a cross-sectional study using data from the Family Medicine Network (FaMe-Network). FaMe-Network is a primary care registration network, affiliated to the Primary Care department of the Radboud University Medical Centre in Nijmegen, the Netherlands [21]. In 2018, this database consists of approximately 32,000 patients from 26 GPs in seven different general practices throughout the Netherlands [22]. The GPs register all encounters with their patients uniformly using the International Classification of Primary Care (ICPC). For each episode of care, defined as ‘all provided care for a specific health problem or illness in an individual during a set time period’, all interventions are registered, including history, physical examination, diagnostic tests, medical advice, referrals and medical correspondence from hospitals. Drug prescriptions are coded with the Anatomical Therapeutical Chemical (ATC) classification. All diagnostic tests are registered using the diagnostic assay coding table from the Dutch College of General Practitioners [23]. All encounters between GPs and their patients are coded with a diagnosis code (i.e. ‘gastroenteritis’) and a reason for encounter code (RFE) (i.e. ‘vomiting’). The RFE is defined as the first complaint(s), symptom(s), or request(s) the patient mentions when consulting the GP.

### Patient inclusion

Patients enlisted in the general practices between 1 and 6-2013 and 1-7-2018 were eligible for inclusion. Based on previous studies, we defined a ‘total population at risk’: elderly, patients suffering from heart failure, and patients with chronic kidney disease [6, 16, 24]. We included all patients aged ≥70, suffering from heart failure (ICPC2-K77), and/or CKD (ICPC2-U99, and/or having an eGFR /creatinin clearance of < 60 ml/min for at least 90 days) [24], and who all used high-risk medication (diuretics (ATC C03*) and/or NSAIDs (ATC M01A*) and/or RAAS-inhibitors (ATC C09*)). We named this group the ‘total population at risk’. [6, 16, 25]

From the total population at risk, we included patients with an encounter (consultation, house visit, consultation by phone) coded with either one of the following RFEs or diagnoses: chills (ICPC2-A02), fever (ICPC2-A03), vomiting (ICPC2 -D10), diarrhoea (ICPC 2-D11), gastrointestinal infection (ICPC2-D70) and gastroenteritis (ICPC2-D73). These encounters had to take place while the patients used the high-risk medication. We named these encounters ‘dehydration-risk’ encounters. A ‘dehydration-risk’ episode was defined as one or more related encounters of one patient about a ‘dehydration-risk’ problem in a set time period.

### Data extraction

Subsequently, for all included patients, we obtained the electronic patient files. In these files, we manually checked the free text, medical correspondence from the hospital (which is included in the general practice database) and lab results in the ‘dehydration-risk’ encounters. For these ‘dehydration-risk’ encounters we answered the following questions:- Was the patient advised to temporarily discontinue or adjust the dosage of the high-risk medication? - Was this advice offered in the first encounter of an episode or in one of the possible follow-up encounters of this episode?- Did a complication occur in the subsequent 3 months after the first encounter of an episode?

A complication was defined as a secondary disease or condition (e.g. AKI or hypotension) that developed in the course of the initial complaint (e.g. gastroenteritis). AKI was defined as a rapid fall in glomerular filtration rate, clinically manifest as an abrupt increase of creatinine serum levels 1.5 times baseline levels which (presumably) occurred in the prior 7 days or an increase of serum creatinine levels of ≥26.5 μmol in the prior 48 h [26]. Hypotension was defined as a decrease in systolic or diastolic blood pressure of ≥20 mmHg.

The assessment if an event should be labeled as a complication was done by SJF. In doubt, SJF and AAU decided in mutual consultation whether or not the event should be labeled as a complication.

### Outcome measures

Our outcome measures were [1] the percentage of patients with high risk of suffering from complications due to a combination of medication use (RAAS-inhibitor and/or diuretic and/or NSAID) and risk factors (age and/or presence of CKD and/or presence of heart failure) [2]; The percentage of high-risk patients who visit the GP with a ‘dehydration-risk’ episode [3]; The percentage of ‘dehydration-risk’ episodes in which high-risk patients were advised to temporarily discontinue their medication [27]; The amount of times a complication occurred in the subsequent 3 months after the initial encounter.

### Statistical analysis

All statistical analyses were performed using SPSS version 25 software. We carried out simple descriptive statistics to identify the population and episode characteristics, the advice rate and the complication rate in the population.

## Results

### Patient characteristics

**Fig. 1:**
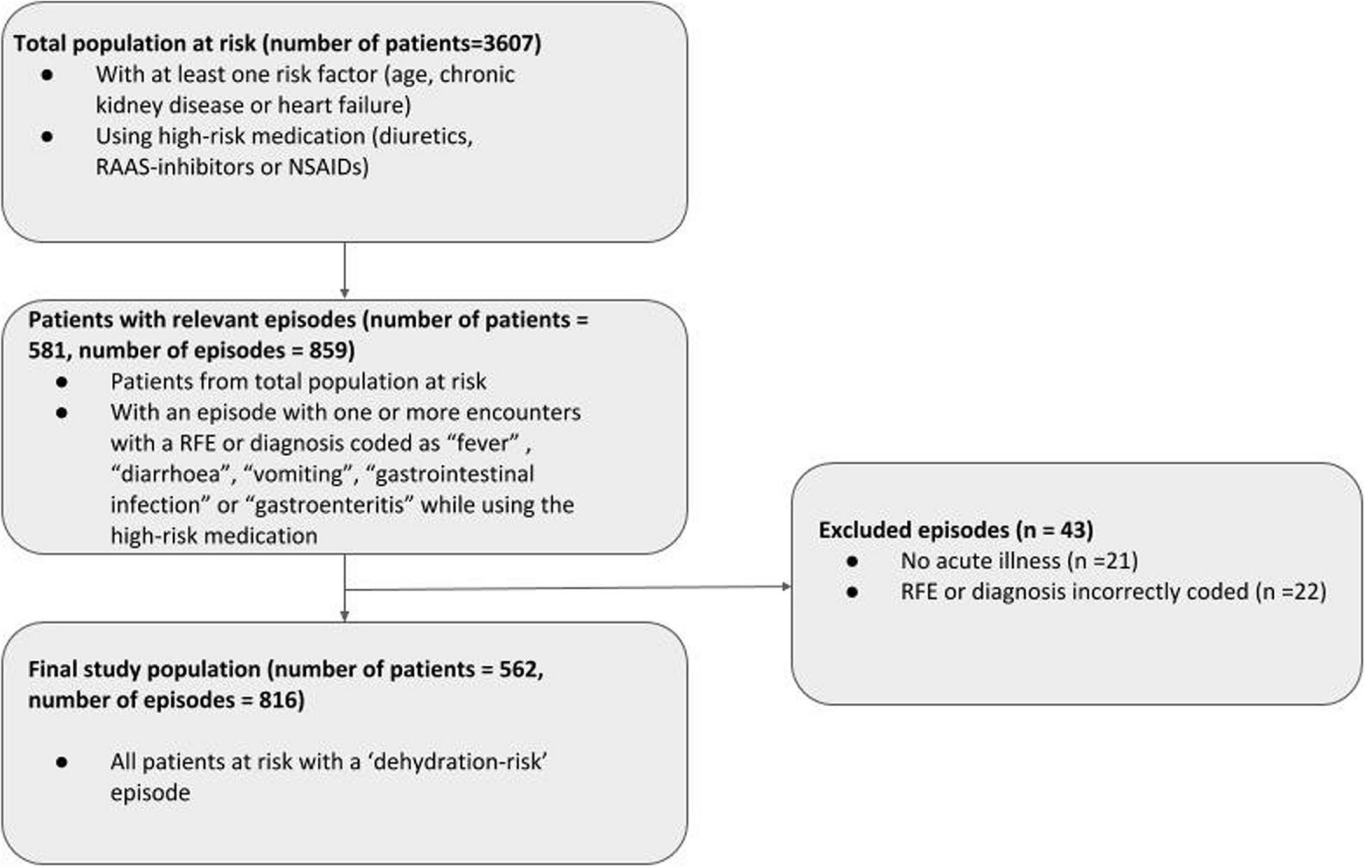
Flowchart of the inclusion process of the final study population

We found 3607 patients from a total of 44.675 different patients (8.1%) who had a high risk of suffering from complications due to a combination of medication use and the presence of the risk factors age, heart failure, or CKD. When adjusted to the time period the patients were registered in the practices, we included 22.6 patients per 1000 patient-years in the ‘total population at risk’. Table 1 shows the characteristics of these patients. From this total population at risk, 581 patients (with a total of 859 episodes) contacted the GP with ‘dehydration-risk’ episodes. After manually checking the free text in these episodes, we excluded 21 episodes with chronic, non-acute complaints, and 22 episodes with incorrectly labeled RFE’s or diagnoses (e.g. episodes labeled with ‘vomiting’, while there was no vomiting, only heartburn). In total, we excluded 19 patients (43 episodes). The final study population comprised of 562 patients with 816 ‘dehydration-risk’ episodes (see Fig. 1). Table 2 shows the characteristics of these episodes. When translating our results to a standard practice of 2095 patients [28], a general practice has on average 167 high risk patients and there will be eight ‘dehydration-risk’ episodes each year.

**Table 1:** Patient characteristics of the total population at risk

Gender (women)	2000 (55.4%)^a^
Medication used^b^	
RAAS-inhibitors	2301 (63.8%)
Diuretics	2005 (55.6%)
NSAIDs	1677 (46.5%)
Combination of all drugs	451 (12.5%)
Risk factors^b^	
Chronic kidney disease	1142 (31.7%)
Heart Failure	665 (18.4%)
Age ≥ 70	3331 (92.3%)
All risk factors	286 (7.9%)

^a^All values are counts (%)

^b^Patients could use more than one drug and could have more than one risk factor

**Table 2:** Contact characteristics for all episodes

Characteristics	All episodes (*n* = 816)
Number of individual patients	562^a^
Age of patients at first contact of episode	79.4 (47–100)^b^
Gender of patients ^c^(women)	333 (59.3%)
Medication used during first contact of episodes^d^	
RAAS-inhibitors	590 (72.3%)
Diuretics	471 (57.7%)
NSAIDs	99 (12.1%)
Combination of all drugs	19 (2.3%)
Most frequent RFEs	
Diarrhoea (D11)	312 (38.2%)
Fever (A03)	245 (30%)
Vomiting (D10)	156 (19.1%)
Most frequent diagnoses	
Diarrhoea (D11)	173 (21.2%)
Gastroenteritis (D73)	133 (16.3%)
Fever (A03)	87 (10.7%)
Risk factors of patients during first contact of episode^d^	
Chronic kidney disease	281 (34.4%)
Heart Failure	239 (29.3%)
Age ≥ 70	728 (89.2%)
All risk factors	100 (12.3%)

^a^All values are counts (%), unless otherwise specified

^b^mean (range)

^c^As proportion of all individual patients (*n* = 562)

^d^Patients could use more than one drug and could have more than one risk factor

### Advice rate

The GP advised to discontinue or adjust the dosage of the high-risk medication in 38 episodes (4.6%). In 28 episodes, this advice was offered in the first encounter of an episode. In the remaining 10 episodes, the advice was offered in one of the follow-up encounters of an episode in which the situation had worsened or remained the same.

In 37 episodes, the GP referred a patient to the hospital (4.5%). In 24 episodes this referral was made in the first encounter of an episode and in 13 episodes in one of the follow-up encounters. In the hospital, the high-risk medication was always temporarily discontinued. In one episode, a patient had already discontinued his diuretic before consulting the GP. In two episodes advice was offered in the first encounter and in one of the follow-up encounters the patient was referred to the hospital because of aggravation of clinical signs. In total, in 743 of 816 episodes (91%) neither advice to discontinue or adjust the dosage of the high-risk medication was offered nor a referral to the hospital was made.

### Complication rate

**Fig. 2:**
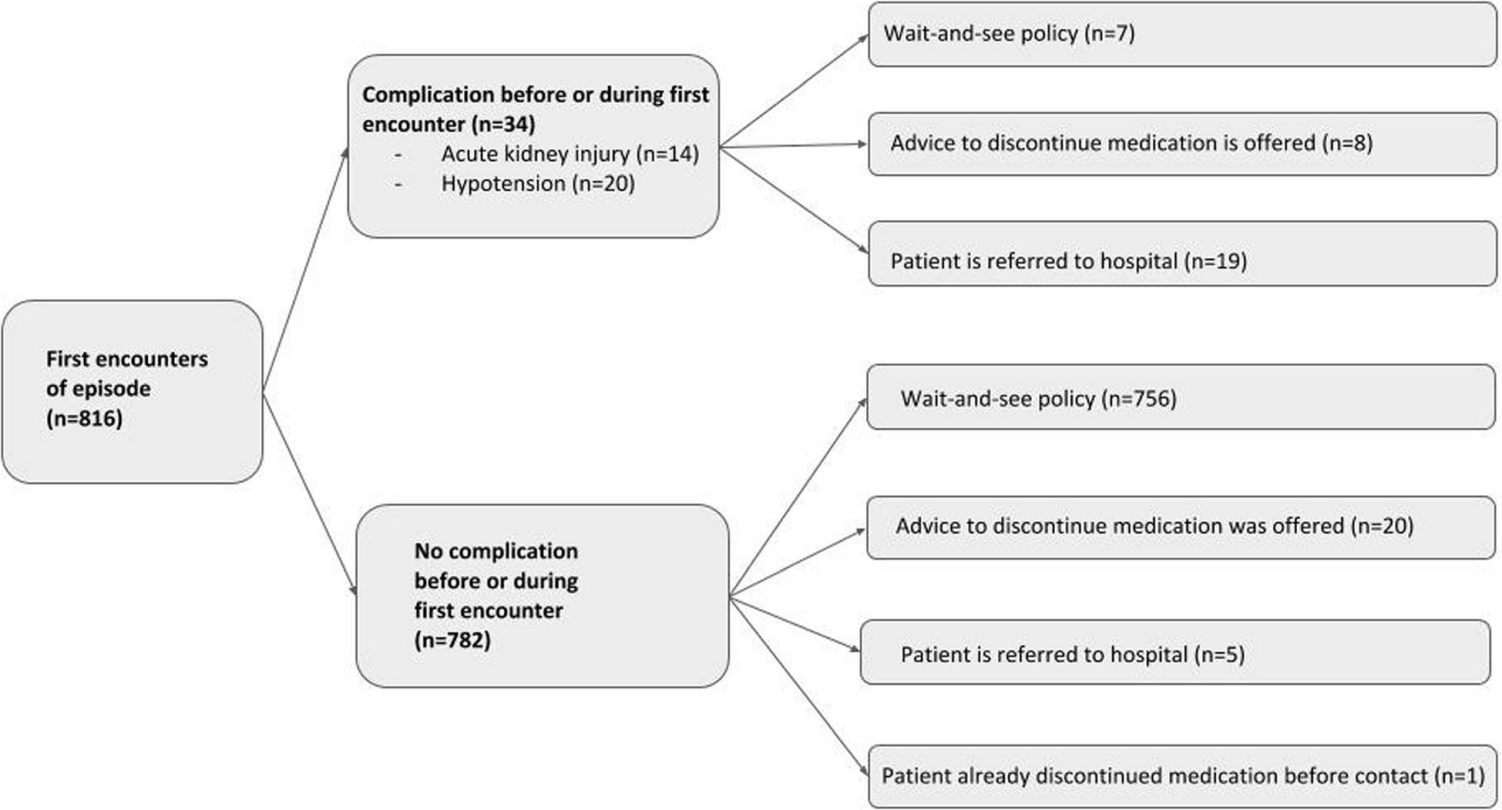
Occurrence of complications and actions by GP in all first encounters of an episode

During 58 of the 816 episodes a total of 59 complications occurred. Figure 2 shows that in 34 (4.2%) episodes, a complication had already occurred before or during the first encounter of the episode. In 19 episodes (AKI (*n* = 14), hypotension (*n* = 5)), the GP referred directly to the hospital. In eight episodes, the GP advised the patient to discontinue the high-risk medication. In seven episodes, the GP applied a wait-and-see policy.

In 25 of 816 episodes (3.1%), a complication occurred in the subsequent 3 months after a patient had contacted the GP. AKI (*n* = 15), hypotension (*n* = 7) and electrolyte disorder (*n* = 3). In 24 of these 25 episodes, the complication occurred in one of the 791 patients thought to be clinically well during the first contact. In one of these 25 episodes, a patient had two complications; one before or during the first encounter and one after the first encounter. In 22 of these 25 (88%) episodes the GP had not given prior advice, regarding the discontinuation or adjustment of the medication. In three episodes, the patients had been advised to discontinue their high-risk medication, but despite this advice AKI (*n* = 2) or hypotension (*n* = 1) occurred. In 7 of these 25 complications, a hospital referral was needed.

In one episode, a complication occurred as a result of adjusting the dosage of the high-risk drug furosemide during a period of diarrhoea. In the days that followed, this patient suffered from dyspnoea and edema which was attributed to decompensatio cordis due to the adjustment of the furosemide dosage.

## Discussion

### Summary of all findings

Approximately 22.6 patients per 1000 patient-years have a high risk of suffering from complications due to a combination of risk factors (age > 70 years, CKD, heart failure) and medication use. In 91% of the 816 analysed ‘dehydration-risk’ episodes, GPs neither advised to adjust the dosage of the high-risk medication nor referred to the hospital.We observed a total of 59 complications in 816 episodes (7.1%). In 34 episodes (4.1%), complications occurred before or during the first encounter with the GP. (AKI (*n* = 14), hypotension (*n* = 20)). In 25 episodes (3.1%), complications occurred after the patient had already contacted the GP (AKI (*n* = 15), hypotension (*n* = 7), electrolyte disorder (*n* = 3)). In 22 of these 25 complications the GP had not given prior advice to discontinue medication.

### Comparison with literature

The prevalence of patients with a high risk of suffering from complications due to a combination of risk factors, medication use and concurrent illness in a general practice has been poorly investigated so far. Scherpbier et al. estimated that in a standard general practice in the Netherlands, approximately 40 patients have CKD and use RAAS-inhibitors at the same time [6]. Our estimations correspond with these findings: we estimated that in a standard practice 167 patients have at least one of the risk factors (age ≥ 70 years, heart failure, or CKD) and simultaneously use a RAAS-inhibitor, NSAID, or diuretic [29]. In total, 41 of those patients suffer from CKD while using RAAS-inhibitors.

The occurrence of complications due to the ‘cocktail’ of dangerous factors like age, medication use, and concurrent illness has been described frequently in case reports [6, 14, 15]. However, the number of complications in general practice remained unclear. In the cohort study performed by Stirling et al. [15] 2398 admissions in a UK hospital were included. Of this cohort, 30 high-risk patients suffered from AKI while using RAAS-inhibitors, six (20%) of these patients were volume depleted due to gastrointestinal complaints. In these six patients, the volume depletion in combination with using RAAS-inhibitors was the probable cause of AKI. These numbers indicate that a significant part of the patients that suffer from AKI use RAAS-inhibitors and are volume depleted. Our study confirms this with a complication rate of 7.1% of all episodes with patients that use RAAS-inhibitors, diuretics, or NSAIDs while being volume depleted.

### Strengths and limitations

The strengths of this study are the relatively long observational period of 5 years and the quality of registration in the FaMe-Network, which is high due to intensive education and training of the participating GPs and doctor’s assistants. Furthermore, we manually checked the medical records in which all notes, lab results, drug prescriptions and medical correspondence are available. This gave us a more detailed view of what happened to the patients instead of only relying on the coded information registered in the FaMe-Network. This study also has limitations. Because of the small numbers of patients in the medication-discontinuation group, we could not statistically compare outcome measures between the group of patients with ‘dehydration-risk’episodes’ who continued their medication and the group who discontinued their medication. Furthermore, in some cases, it was hard to establish whether or not a complication had occurred, or advice was offered. This was due to the fact that some notes by the GP or doctor’s assistants were very brief. For example, if blood pressure was not measured, it was unknown whether hypotension occurred. Moreover, it is possible that patients experience only few symptoms, that improve over time, and therefore do not encounter their GP. Another limitation is the small amount of practices analysed, which lowers the generalisability. The prevalence of patients with CKD and the percentage of elderly (≥70 years) in the included practices is significantly lower (1.5 and 8.5% respectively) than in the overall Dutch population (12 and 23% respectively) [20, 28]. Therefore, we expect even more high-risk patients and more complications in general Dutch population.

### Implications for future research

An important subject for future research is to analyse the cause of the current low advice rate. This low advice rate could be explained by an insufficient awareness of the GP regarding the risks of continuing high-risk medication during fluid loss. Another explanation could be that GPs fear complications due to the discontinuation of high-risk medication or that GPs consciously decide not to discontinue medication because of results of physical investigation.

### Clinical impact

We calculated that, in a standard Dutch General practice, approximately eight high-risk patients each year contact the GP for a complaint in which – according to the guidelines - the GP should advise the patient to (temporarily) discontinue their high-risk medication in order to prevent complications. Currently, in 91% of all episodes neither advice is offered, nor a referral is made. We do not know in what percentage the GP did consider offering the advice, but consciously decided to continue medication. In the free text, we found 10 episodes in which the GP noted the reason for continuing the high-risk medication. It is possible that GPs made this consideration more often, but did not write this down in the medical record.

The overall complication rate of 7.1% means that in every 14 ‘dehydration-risk’ episodes in high-risk patients one complication occurs (58 complications in 816 episodes, so 1 complication in 14 episodes). We believe this is a significant amount with a high clinical importance. A part of these complications could possibly have been prevented when the GPs advise their patients to temporarily discontinue high-risk medication during times of fluid loss and a part of these complications could have been prevented by clear patient instructions at the moment of the first prescription of the high-risk medication.

Therefore, we recommend education in this field for GPs and their assistants and clear instructions for patients when to temporarily discontinue high-risk medication.

## Conclusion

Dutch GPs do not frequently advise high-risk patients to discontinue high-risk medication during ‘dehydration-risk’ episodes. Unfortunately, complications such as acute kidney injury do occur. Timely discontinuation of high-risk medication needs more attention in general practice in order to prevent drug-related complications.

## Data Availability

The datasets used and/or analysed during the current study are available from the corresponding author on reasonable request. https://bronnen.zorggegevens.nl/Bron?naam=Family-Medicine-Network

## Abbreviations

AKI: Acute kidney injury
ATC: Anatomical Therapeutical Chemical
CKD: Chronic kidney disease
FaMe-Network: Family Medicine Network
GP: General practitioner
ICPC: International Classification of Primary Care
NSAID: Non-steroidal anti-inflammatory drug
RAAS: Renin-angiotensin-aldosterone-system
RFE: Reason For Encounter

## References

[1] Lavizzo-Mourey RJ (1987). Dehydration in the elderly: a short review. J Natl Med Assoc.

[2] Canavan A, Arant BS (2009). Diagnosis and management of dehydration in children. Am Fam Physician.

[3] Goulet ED (2012). Dehydration and endurance performance in competitive athletes. Nutr Rev.

[4] National Clinical Guideline C (2013). National Institute for Health and Clinical Excellence: Guidance. Acute Kidney Injury: Prevention, Detection and Management Up to the Point of Renal Replacement Therapy.

[5] Kreimeier U (2000). Pathophysiology of fluid imbalance. Crit Care.

[6] Scherpbier ND, de Grauw WJ, Wetzels JF, Vervoort GM (2010). Acute renal failure due to RAAS-inhibitors combined with dehydration. Ned Tijdschr Geneeskd.

[7] van Geffen K (2016). Nieren en medicijnen. Nierstichting.

[8] Luciano R. NSAIDs: Acute kidney injury (acute renal failure). In: Palevsky PM, editor. Uptodate Waltham, MA. https://www.uptodate.com/contents/nsaids-acute-kidney-injury-acute-renal-failure. Last update March 2017. Accessed 1 Sept 2018.

[9] Sraer JD, Kanfer A, Rondeau E, Lacave R (1989). Role of the renin-angiotensin system in the regulation of glomerular filtration. J Cardiovasc Pharmacol.

[10] Burnier M (2016). Blockade of the renin-angiotensin system and the risk of acute kidney injury. J Clin Hypertens (Greenwich).

[11] Dunn MJ (1989). Prostaglandin I2 and the kidney. Archives des maladies du coeur et des vaisseaux.

[12] Erdbruegger U. Etiology and diagnosis of prerenal disease and acute tubular necrosis in acute kidney injury in adults. In: Palevsky PM, editor. Uptodate Waltham, MA. https://www.uptodate.com/contents/etiology-and-diagnosis-of-prerenal-disease-and-acute-tubular-necrosis-in-acute-kidney-injury-in-adults. Last updated May 2018. Accessed 1 Sept 2018.

[13] Che ML, Yan YC, Zhang Y, Gu Y, Wang NS, Chen N (2009). Analysis of drug-induced acute renal failure in Shanghai. Zhonghua Yi Xue Za Zhi.

[14] McMurray J, Matthews DM (1987). Consequences of fluid loss in patients treated with ACE inhibitors. Postgrad Med J.

[15] Stirling C, Houston J, Robertson S, Boyle J, Allan A, Norrie J (2003). Diarrhoea, vomiting and ACE inhibitors:--an important cause of acute renal failure. J Hum Hypertens.

[16] Baraldi A, Ballestri M, Rapana R, Lucchi L, Borella P, Leonelli M (1998). Acute renal failure of medical type in an elderly population. Nephrol Dial Transplant.

[17] Wynckel A, Ebikili B, Melin JP, Randoux C, Lavaud S, Chanard J (1998). Long-term follow-up of acute renal failure caused by angiotensin converting enzyme inhibitors. Am J Hypertens.

[18] Chaumont M, Pourcelet A, van Nuffelen M, Racape J, Leeman M, Hougardy JM (2016). Acute kidney injury in elderly patients with chronic kidney disease: do angiotensin-converting enzyme inhibitors carry a risk?. J Clin Hypertens (Greenwich).

[19] Belo J, Bos M, Brühl F, Lemmen W, Pijpers M, van den Donk M (2014). NHG-Standaard Acute diarree (derde herziening). Huisarts Wet.

[20] De Grauw W, De Leest K, Schenk P, Scherpbier-De Haan N, Tjin-A-Ton J, Tuut M, Van Balen J, NHG-Standaard Chronische nierschade, 2018. Available from: www.nhg.org/standaarden.

[21] Family Medicine Network (FaMe-net) Available from: https://www.transhis.nl/language/nl/

[22] Family Medicine Network (FaMe-net), deelnemers transitieproject. Available from: https://www.transhis.nl/over/deelnemers-transitieproject/.

[23] NHG diagnostische bepalingen. Available from: https://www.nhg.org/themas/publicaties/nhg-tabel-diagnostische-bepalingen. Accessed Sept 2018.

[24] KDOQI Clinical Practice Guideline for Diabetes and CKD (2012). 2012 Update. Am J Kidney Dis.

[25] Ahmed A (2002). Use of angiotensin-converting enzyme inhibitors in patients with heart failure and renal insufficiency: how concerned should we be by the rise in serum creatinine?. J Am Geriatr Soc.

[26] Khwaja A (2012). KDIGO clinical practice guidelines for acute kidney injury. Nephron Clin Pract.

[27] Uitdroging ouderen. Available from: https://www.thuisarts.nl/uitdroging/ik-wil-uitdroging-bij-ouderen-voorkomen. Accessed 5 Sept 2018.

[28] LHV Kerncijfers Huisartsenzorg. Available from: https://www.lhv.nl/uw-beroep/over-de-huisarts/kerncijfers-huisartsenzorg. Accessed Oct 2018.

[29] Centraal Bureau voor de Statistiek Bevolking; geslacht, leeftijd en burgerlijke staat 1 januari 2017.

